# Knowledge and Attitudes on Preventing and Treating Dentin Hypersensitivity and Its Predicting Factors: A Cross-sectional Study with Brazilian Citizens

**DOI:** 10.1055/s-0042-1757905

**Published:** 2022-12-13

**Authors:** Victor Mosquim, Gabriela Utrago Carneiro, Gerson Aparecido Foratori-Junior, Heitor Marques Honório, David Geoffrey Gillam, Linda Wang

**Affiliations:** 1 Department of Operative Dentistry, Endodontics and Dental Materials, Bauru School of Dentistry, University of São Paulo, Bauru, São Paulo, Brazil; 2 Department of Pediatric Dentistry, Orthodontics and Public Health, Bauru School of Dentistry, University of São Paulo, Bauru, São Paulo, Brazil; 3 Centre for Oral Bioengineering, Institute of Dentistry, Barts and The London School of Medicine and Dentistry, Queen Mary University of London, London, United Kingdom

**Keywords:** dentin hypersensitivity, patient, health, questionnaire, regression analysis, surveys

## Abstract

**Objectives** Most dental schools have included dentin hypersensitivity (DH) as part of their taught curriculum to educate undergraduates; however, it is possible that the public still does not recognize its symptoms and the factors that predispose to the onset of this condition. Thus, the aim of this study was to assess the knowledge of a Brazilian population regarding the prevention and treatment of DH and to identify what self-reported factors can serve as predictors of the frequency of DH.

**Materials and Methods** An online questionnaire investigated the demographic characteristics, oral health self-perception and attitudes, and DH prevention and treatment measures of 226 participants.

**Statistical analysis** Data were analyzed descriptively and by a multiple linear regression with DH frequency score as the dependent variable (α < 0.05).

**Results** Total 61.1% of females ( *n* = 138) and 38.9% of males ( *n* = 88) (mean age: 35.1 ± 12.2 years) completed the questionnaire. The sample's mean DH frequency score (minimum 0; maximum 20) was 4.2 and classified as low, with 19.1% using desensitizing products and 22.1% reporting having noncarious cervical lesions (NCCLs). When experiencing DH episodes, 21.2% never and 30.1% rarely scheduled dental appointments. Regression analysis retrieved a significant final model ( *F* [5,220] = 12.047; *p* < 0.001; *R* ^2^ = 0.215).

**Conclusion** This study identified that 36.7% and 18.6% of the sample were unaware that DH can be both prevented and treated, respectively. Moreover, the presence of NCCLs, frequency of daily toothbrushing, use of desensitizing products, presence of DH modulating factors, and the presence of parafunctional habits symptoms served as predictors of DH frequency.

## Introduction

Due to its painful characteristics, dentin hypersensitivity (DH) has been shown to impact on the patient's quality of life. 1 A systematic review estimated that the prevalence of DH is 11.7% worldwide and affects more women and young adults. 2 3 Yet, despite the importance of preventing and treating this condition, studies reported that dentists commonly control its symptoms without, in fact, controlling its etiological factors. 4 5 6

Previous studies on the knowledge of dentists about the management of DH have been published. 4 5 6 7 8 9 10 For example, Zeola et al 5 reported that dentists are already attentive and concerned about controlling the predisposing factors for the development of DH, although Francisconi-dos-Rios et al 6 and Exarchou et al 9 reported that the professionals' and students' knowledge on this topic still required improvement. Moreover, although several dental schools have included teaching on DH and noncarious cervical lesions (NCCLs) in their curricula, communication with the local lay community was often neglected by the profession. In these cases, even though the dentist can perform the treatment, patients do not seek dental care because they either do not identify this condition as treatable or consider the condition as a relatively minor inconvenience not requiring treatment. 4 9

Several factors have also been reported to be associated with DH, such as orthodontic treatment, frequency of daily toothbrushing, use of dietary supplements by athletes, gastroesophageal disorders, use of illicit drugs, and even parafunctional habits. 11 12 13 14 15 16 Some of these factors are daily habits unknown or overlooked by the population. Therefore, to practice person-centered care, the patient should be made aware of these habits to make the process of prevention and treatment easier, simpler and to reduce the costs related to the maintenance of oral health. 17 Thus, it is necessary to identify what self-reported habits can serve as predictors for DH for dentists to modify or eliminate these factors when considering the prevention and treatment of the condition.

Therefore, the aim of this study was to assess the knowledge of a sample of the Brazilian population regarding prevention, diagnosis, and treatment of DH, and to identify what self-reported factors are associated with it and can serve as predictors of the presence of DH.

## Materials and Methods

The CHERRIES (Checklist for Reporting Results of Internet E-Surveys) 18 and the STROBE (Strengthening the Reporting of Observational Studies in Epidemiology) 19 guidelines were used to report this observational cross-sectional study.

### Study Design and Ethical Aspects

This study was conducted using a voluntary online questionnaire open survey with a convenience sample. The questionnaire was specifically developed for this study and was composed by sociodemographic questions, followed by 22 questions about DH and its etiological factors. This study was performed following approval by the local Human Research Ethics Committee (CAAE: 46103021.3.0000.5417).

### Development and Testing

The questionnaire was developed by two researchers based on the complaints and on the clinical signs and symptoms detected by dentists during the care of patients with DH at the university dental clinics.

After developing it, the questionnaire was revised by independent experts regarding its content and clarity (content validity analysis). These experts reported to the developers what questions were not clear and/or irrelevant, so the questionnaire could be modified until all the questions were unanimously considered clear and relevant.

Then, the questionnaire usability and technical functionality were pretested with a small sample composed of lay citizens (not related to the dentistry field) and dentists using different electronic devices. Considering their answers and feedback, the questionnaire was once again adjusted to better fit the aims of the authors and to improve its clarity, comprehensiveness, and acceptability. Thereafter, due to the modifications made to the questionnaire after the pretest, the data generated during the pretest were discarded before the commencement of the study.

### Questionnaire Characteristics

The questionnaire was developed through the Google Forms platform. Before answering, the participants were educated about DH and informed that the estimated time required to complete all questions was 10 minutes. The participants were also informed on the purpose of the study, who were the investigators, and that their answers and e-mail addresses would be stored only for the purpose of the study. Questionnaire responses were kept confidential, and participants were kept anonymous, but an e-mail account was necessary to prevent duplicate entries. No cookies nor IP checks were used. No incentives were offered, and data were kept secure in a Google Drive account, which could be accessed only by this study's investigators.

The questionnaire (unvalidated English version available as Appendix 1) was structured in four main pages: (1) the first page involved six sociodemographic questions, (2) the second page (with seven questions) addressed the participants' perception of their own oral health (with pictures available for consultation); (3) the third page (two questions) included specific questions related to the impact of other dental treatments on DH; and (4) the final page (seven questions), specific questions about DH prevention and treatment.

All questions were set as mandatory, and the questionnaire could not be submitted without its completion. The questions and the alternatives were not randomized for each participant and no consistency or integrity check was performed before the questionnaire was submitted. The participants were able to revise and, if necessary, modify their answers before submission.

### Sample Size Calculation

The sample size was estimated according to Faul et al, 20 whose model containing *f* ^2^ = 0.15, α error = 0.05 and power (1 − β) = 0.95 estimated that a sample with *n* = 178 was necessary when considering 11 independent variables inserted in a multiple linear regression model.

### Sample Acquisition

Social media were used to recruit participants from all regions of Brazil. The posts and invitations contained a description of the study and the link to access the questionnaire.

In total, 227 people accessed the questionnaire; however, one person did not consent to participate in the study, so the sample consisted of 226 participants who completed the questionnaire between July and December 2021.

### Eligibility Criteria

The sample was composed of Brazilian citizens from all parts of Brazil who were at least 18 years old and did not graduate as dentists or were currently enrolled in a dentistry course. Individuals without a resident visa in Brazil were not included in the sample.

### Data Interpretation

Data interpretation(s) were comparable to the methodological interpretation used by Foratori-Junior et al, 21 for example, the educational level was categorized from 0 to 5 as: 0, illiteracy; level 1, incomplete high school; level 2, complete high school; level 3, incomplete higher education; level 4, complete higher education; and level 5, complete postgraduation degree.

Monthly household income was organized into six categories according to the minimum wage (MW) approved by the Brazilian government for the year 2021 as R$ 1,100.00 (approximately USD 199): level 1, up to 1 MW; level 2, between 1 and 2 MW; level 3, between 2 and 3 MW; level 4, between 3 and 4 MW; level 5, between 4 and 5 MW; and level 6, more than 5 MW.

All answers to the questions that evaluated the frequency with which a certain situation occurred were ordered in a modified Likert scale, with score 0 assigned to the answers “never”; 1 to “rarely”; 2 to “sometimes”; 3 to “often”; and 4 to “always.” Based on these scores, the answers to the questions related to the frequency with which the participants experienced DH when (1) ingesting cold water, (2) talking in windy environments, (3) ingesting a cold drink, (4) ingesting acidic foods, and (5) brushing their teeth were added and grouped in the variable “frequency of DH,” with a minimum score of 0 and a maximum of 20. The “frequency of DH” score was categorized into nonexistent (score 0), low (between 1 and 6), moderate (between 7 and 13), and high (between 14 and 20).

Similarly to that stated earlier, the score of the questions related to the frequency with which the participant (1) consumed acidic foods or drinks, (2) felt stressed or anxious, (3) gritted teeth, (4) pressed tongue against teeth, (5) bit lips, cheeks, or tongue, (6) chewed on objects (such as pens, glasses, or nail), (7) brushed their teeth vigorously, and (8) brushed their teeth with a medium-bristled toothbrush were summarized and grouped in the variable “modulating factors,” with a minimum score of 0 and a maximum of 32. The score was also categorized into nonexistent (score 0), low (between 1 and 10), moderate (between 11 and 20), and high (between 21 and 32).

The score for the answers to questions related to the frequency with which the participants reported (1) difficulties opening their mouth upon waking up, (2) headaches, (3) pain or tension in the neck, and/or (4) pain near the ears were added and grouped in the variable “symptom of parafunction,” with a score minimum of 0 and maximum of 16. The score was categorized into nonexistent (score 0), low (between 1 and 5), moderate (between 6 and 10), and high (between 11 and 16).

### Statistical Analysis

Data were organized and a qualitative descriptive analysis was performed, expressing the data in absolute numbers, percentages, and means. Also, the participants' answers for variables “frequency of DH,” “modulating factors,” and “symptom of parafunction” were subjected to a reliability analysis using Cronbach's alpha test.

A multiple linear regression analysis was performed (backward method), in which the dependent variable was the “frequency of DH” (score between 0 and 20). The analysis of the residues was performed, and the independency of the residues, presence of outliers, normal distribution, multicollinearity, and homoscedasticity were checked. A significance level of 5% was adopted. The independent variables included in the regression model were gender, age, educational level, household monthly income, presence of NCCLs, presence of carious lesions, frequency of daily toothbrushing, use of orthodontic appliances, use of a product for DH, modulating factors, and symptom of parafunction.

All tests were conducted using the IBM SPSS Statistics v25 (IBM Corp, Armonk, New York, United States) software.

## Results

All 226 participants were residents of the Northeast, Midwest, South, and Southeast regions of Brazil and presented ages between 18 and 74 years (mean: 35.1 ± 12.2). The sociodemographic characteristics are displayed in Table 1 . The Cronbach's alpha test retrieved a value of α = 0.726, therefore was considered acceptable.

**Table 1:** Sample sociodemographic characteristics ( *n* = 226)

Sociodemographic	*n*	%
Gender		
Females	138	61.1
Males	88	38.9
Educational level		
Illiterate	0	0
Incomplete high school	4	1.8
Complete high school	25	11
Incomplete higher education	51	22.6
Complete higher education	58	25.7
Postgraduation degree	88	38.9
Household monthly income		
Less than 1 minimum wage	27	11.9
Between 1 and 2 minimum wages	33	14.6
Between 2 and 3 minimum wages	32	14.2
Between 3 and 4 minimum wages	20	8.9
Between 4 and 5 minimum wages	26	11.5
More than 5 minimum wages	88	38.9
Dental care		
In the private sector	192	84.9
In the public sector	16	7.1
In both sectors	18	8

As for the “frequency of DH” score ( Table 2 ), the mean value among the participants was 4.2; therefore, it was classified as low. The situations that the participants reported triggering DH episodes more frequently were, respectively, when (1) ingesting cold foods, (2) brushing their teeth, (3) ingesting some hot food, (4) ingesting acidic foods, and (5) speaking in windy environments.

**Table 2:** Participants' self-perception of oral health and previous dental treatments

Self-perception of oral health and previous dental treatments	*n*	%
Frequency of DH scores		
Nonexistent (score 0)	22	9.8
Low (scores 1–6)	158	69.9
Moderate (scores 7–13)	43	19
High (scores 14–20)	3	1.3
Modulating factors scores		
Nonexistent (score 0)	0	0
Low (scores 1–10)	58	25.7
Moderate (scores 11–20)	148	65.5
High (scores 21–32)	20	8.8
Parafunction symptom scores		
Nonexistent (score 0)	10	4.4
Low (scores 1–5)	133	58.8
Moderate (scores 6 to 10)	70	31
High (scores 11–16)	13	5.8
Presence of carious lesions		
Yes	60	26.5
No	120	53.1
Do not know	46	20.4
Presence of NCCLs		
Yes	50	22.1
No	133	58.9
Do not know	43	19
Used product for DH		
Yes	43	19
No	182	80.5
Do not know	1	0.5
Frequency of daily toothbrushing		
Do not brush their teeth	0	0
Once a day	9	4
Twice a day	62	27.4
Thrice a day	108	47.8
More than three times a day	47	20.8
Using/used orthodontic appliances		
Yes	162	71.7
No	64	28.3

Abbreviations: DH, dentin hypersensitivity; NCCL, noncarious cervical lesion.

Most of the sample also reported not having carious lesions nor NCCLs ( Table 2 ). Also, 80.5% of the participants reported not using any DH product. Among the participants who reported using a product for DH, 79.1% ( *n* = 34) used it after being prescribed by a dentist, 7% ( *n* = 3) used products recommended by friends or family, 7% ( *n* = 3) by advertisements on television or social media, and 7% ( *n* = 3) by other communication vehicles.

When asked about the frequency with which they used DH products, 64.6% ( *n* = 146) reported never using these products without their dentist's instructions, 15.9% ( *n* = 36) reported doing so rarely; 10.2% ( *n* = 23) sometimes; 7.1% ( *n* = 16) frequently; and 2.2% ( *n* = 5) always. However, when asked about the frequency with which they scheduled an appointment with a dentist following experiencing DH episodes, 21.2% ( *n* = 48) reported never doing it; 30.1% ( *n* = 68), rarely; 23.9% ( *n* = 54), sometimes; 15% ( *n* = 34), frequently; and 9.7% ( *n* = 22), always.

The results for the frequency of daily toothbrushing and use of orthodontic appliances are displayed in Table 2 . Of those participants who wore orthodontic appliances ( *n* = 162), 83.9% ( *n* = 136) did not use any product for DH; 15.4% ( *n* = 25) used at least one product for DH, and 0.6% ( *n* = 1) did not know if the product they used was for DH. On the contrary, those participants who never wore orthodontic appliances ( *n* = 64), 71.9% ( *n* = 46) did not use any product for DH, whereas 28.1% ( *n* = 18) did. The frequency of DH score of those who wore orthodontic appliances and of those who never did were both 4.2 and classified as low.

When the modulating factors were measured ( Table 2 ), the mean value among the participants was 14.2. Hence, the frequency of DH scores of the sample was classified as moderate. Habits that patients reported more frequently were, respectively, (1) consumption of acidic foods, (2) feeling stressed and/or anxious; (3) grinding or clenching teeth; (4) brushing teeth with force; (5) biting lips, cheeks, or tongue; (6) pressing the tongue against the teeth; (7) chewing on objects; and (8) using a toothbrush with medium or hard bristles.

In respect of parafunctional habits ( Table 2 ), the mean value among the participants was 4.8; therefore, it was classified as low. The symptoms that patients reported most frequently were as follows: (1) neck pain or tension, (2) headaches, (3) pain near the ears, and (4) difficulty in opening the mouth upon waking up.

Overall, 63.3% of the participants answered they acknowledged DH could be prevented ( Table 3 ), and 81.4% reported knowing that DH could be treated. Also, 60.2% believed that DH treatment is temporary in nature. The participants' answers for the use of toothpastes, laser, orthodontic appliances, and professional products on the treatment of DH are displayed in Table 3 .

**Table 3:** Participants' answers about DH prevention and treatment

DH prevention and treatment	*n*	%
Knew that DH can be prevented		
Yes	143	63.3
No	83	36.7
Knew that DH can be treated		
Yes	184	81.4
No	42	18.6
The treatment is		
Definitive	32	14.2
Temporary	136	60.2
I do not know	58	25.6
Can toothpastes be used in the treatment of DH		
Yes	197	87.2
No	8	3.5
I do not know	21	9.3
Can lasers be used in the treatment of DH		
Yes	56	24.8
No	31	13.7
I do not know	139	61.5
Can orthodontic appliances be used in the treatment of DH		
Yes	32	14.2
No	88	38.9
I do not know	106	46.9
Can professional products be used in the treatment of DH		
Yes	188	83.2
No	8	3.5
I do not know	30	13.3

Abbreviation: DH, dentin hypersensitivity.

Regarding the multiple linear regression, multicollinearity (tolerance values >0.772; Variance Inflation Factor (VIF) values <1.295) and outliers were not detected, and the residuals were independent (Durbin–Watson <1.899). This analysis resulted in a statistically significant final model ( *F* [5,220] = 12.047; *p* < 0.001; *R* ^2^ = 0.215) with the independent variables (1) presence of NCCLs, (2) frequency of daily toothbrushing, (3) use of any product for DH, (4) modulating factors, and (5) symptoms of parafunction present in the final model as possible predictors of the dependent variable ( Table 4 ).

**Table 4:** Final multiple linear regression model indicating the predictive variables related to the dependent variable

Dependent variable	Independent variable	Standardized β	*t*	*p* -Value
Frequency of DH	–	–	–	–
	Presence of NCCLs	0.126	2.092	0.038
	Frequency of daily toothbrushing	−0.101	−1.667	0.097
	Use of desensitizing product	0.262	4.334	<0.001
	Modulating factors	0.220	3.231	0.001
	Symptom of parafunction	0.168	2.497	0.013

Abbreviation: DH, dentin hypersensitivity, NCCLs, noncarious cervical lesions.

## Discussion

This study identified that 36.7% and 18.6% of the population did not know that DH can be prevented or treated, respectively. Moreover, despite the participants having a low frequency of DH episodes and symptoms of parafunctional habits, the presence of habits that can lead to the development of DH was moderate in nature. Finally, the study also reported on some of the predictors of the frequency with which patients experienced episodes of DH, such as: (1) presence of NCCLs, (2) frequency of daily toothbrushing, (3) use of products for DH, (4) presence of modulating factors, and (5) parafunctional habits symptoms.

As reported by Gillam et al, 22 DH can only be diagnosed by excluding other potential causes for dental pain. Therefore, the information collected during screening and clinical examination stages are essential to exclude other conditions with similar pain characteristics, including dental caries, pulpitis, molar–incisor hypomineralization, fractured/chipped restorations/teeth, and gingival inflammation. 13 22 23 24 25 26 In the present study, the presence of carious lesions did not remain in the final regression model, which also indicated that patients were relatively able to differentiate the presence of carious lesions from the symptoms of DH, also validating the final regression model. Yet, the results must be interpreted with caution, given that this is a self-reported study and that patients often consider that they have carious lesions because of a stained area in their teeth while ignoring areas of white spot lesions.

Furthermore, as suggested by other previous studies, 27 28 29 despite DH being a symptom arising from the exposure of dentin to the oral environment and NCCLs being considered a structural defect, these two conditions are closely related. Que et al 27 reported that 15% of the evaluated teeth had NCCLs, while 4.7% had DH. However, 63.3% of the teeth with DH also presented NCCLs, indicating the close relationship between these two conditions. This relationship is relevant since both NCCLs and DH share some etiological factors, 9 27 30 and the presence of NCCLs often lead to exposure of dentin, which, in turn, may lead to the onset of DH. This justifies the presence of NCCLs being a predictive factor in the final regression model in the present study ( *p* = 0.038). This finding agrees with other published studies, where an association between DH and NCCLs was also observed. 27 28 31

In a systematic review, 32 the worldwide prevalence of NCCLs is estimated at 46.7%, contrasting with the 22.1% observed in the present study. Nevertheless, as previously mentioned, data collected in this study were self-reported, and 19% of the sample could not indicate whether they had any type of NCCL, even though NCCLs images were available during the completion of the questionnaire. This suggests that, if NCCLs were identified during a clinical examination, the prevalence of NCCLs in the present study could have been closer to that reported in the systematic review.

Previous studies also reported an association between dental erosion and DH. 9 15 33 In the present study, the presence of habits related to the consumption of acidic foods or drinks presented the highest frequency values between the modulating factors of DH. This observation agreed with the studies of Exarchou et al 9 and O'Toole and Bartlett, 15 in which patients with erosive eating habits had a higher prevalence of DH symptoms ( *p* < 0.001) and corroborated the fact that the independent variable “modulating factors” remained as a predictor in the final regression model ( *p* = 0.001) in the present study.

However, although an acidic diet may be an etiological factor for DH, 9 15 22 33 some associated habits appeared to increase this loss, such as the use of abrasive toothpastes 33 34 35 and the habit of sipping or holding the drink in the mouth for several seconds. 15 Brushing frequency and force, as well as brush stiffness, are also able to act as cofactors in the development of NCCLs, 30 36 which justifies the fact that the brushing frequency remained in the final regression model in this study. However, as described by O'Toole and Bartlett, 15 the frequency of brushing appeared to have a lower relevance for the development of DH than the stiffness of the brush bristles and the use of abrasive toothpastes, which may explain the fact this variable did not show statistical significance within the final regression model ( *p* = 0.097).

When considering the importance of this condition, it is important that desensitizing toothpastes are recommended by a dentist. In the present study, although 87.2% of the participants stated knowing that toothpastes could be used to control DH and 19% used a desensitizing product, 20.9% of the participants reported that the products they used had not been recommended by a dentist. These data agree with the conclusions by Medeiros et al, 28 in which an association was detected between the use of desensitizing toothpastes and the presence of NCCLs, and with Gillam et al, 4 where 23.3% of participants reported using a desensitizing dentifrice. These data are particularly important to the present study because the use of desensitizing products was one of the predictive variables in the final regression model ( *p* < 0.001).

The desensitizing product that the participants reported knowing the most about was a toothpaste, with the use of lasers being the least known. This is probably due to the easy access of this sample population to the former, but it is also possible that the lack of knowledge of other resources is either related to the lack of communication between the dentist and the patient or to the fact that only 15% and 9.7% of patients frequently and always, respectively, scheduled an appointment with a dentist upon experiencing a DH episode. This observation agrees with a previous study, where 48% of patients reporting sensitivity never consulted a dentist. 37 Other published studies have reported that DH is not perceived as a severe problem 9 ; therefore, patients do not seek treatment, 4 making it particularly difficult to manage the symptoms and educate them.

Moreover, contrary to the study of Medeiros et al, 28 the use of orthodontic appliances did not remain as a predictive factor in the final regression model of the present study. However, the relationship between orthodontic treatment and DH is widely discussed in the published literature, as there is evidence that the use of orthodontics in patients with gingival recession due to trauma appears to reduce gingival recession following bone remodeling during tooth movement. 38 Conversely, there is also evidence that orthodontic movement increases the prevalence of gingival recession in patients with anteversion of the lower anterior teeth and in patients requiring maxillary arch expansion. 39 Future studies are therefore required to investigate the association of orthodontic movement with DH. Thus, as orthodontic movement can be beneficial or harmful for gingival recession depending on its causal factor, and while the causal factor of recession was not investigated in each participant, it is possible that these factors were canceled out, justifying the absence of an association with the frequency of DH in this study.

The present study also has its limitations, for example, the questionnaire was based on self-reported answers, and therefore, the participants could have had different interpretations of a particular question, which in turn could influence their answers. Furthermore, despite the questionnaire being answered anonymously, the existence of the Hawthorne effect cannot be ignored, especially in questions related to (1) the frequency of daily toothbrushing, (2) if the participants scheduled an appointment with the dentist following experiencing an DH episode, and (3) if they used products with their dentist's advice.

In addition, the cross-sectional model and the representativeness of the sample are also limited, since most study participants had an average household monthly income more than 5 MW, which differs from the data reported by the Brazilian Institute of Geography and Statistics (Instituto Brasileiro de Geografia e Estatística [IBGE]), where the average income of the Brazilian population in 2021 was close to 2 MW. 40

Despite these limitations, understanding the population's knowledge on DH is also important to practice the person-centered care approach, 17 as this helps professionals to understand which habits may be related to onset of the disease and what information helps the patient to become an active part in their own treatment. This approach may therefore help make the process of prevention and treatment easier and cheaper, which increases the possibility of a longer lasting result.

## Conclusion

In conclusion, 36.7% of a Brazilian population sample were unaware that DH can be prevented, whereas 18.6% were also unaware that DH can be treated. Furthermore, the presence of NCCLs, frequency of daily toothbrushing, use of products for DH, presence of DH modulating factors, and presence of symptoms of parafunctional habits were identified as predictive factors of the frequency of DH episodes.
