# Supplementary material for: Knowledge and Attitudes on Preventing and Treating Dentin Hypersensitivity and Its Predicting Factors: A Cross-sectional Study with Brazilian Citizens
Source: Eur J Dent. 2022 Dec 13;17(3):855–62. doi: 10.1055/s-0042-1757905 (PMC10569857; doi:10.1055/s-0042-1757905)
Supplement: Supplementary file 1 — Supplementary Material [file 10-1055-s-0042-1757905-s2242069.pdf]

Appendix 1 The complete questionnaire filled by the participants included in this study.

Appendix 1

COMPLETE QUESTIONNAIRE

I- DEMOGRAPHIC DATA

1. **Gender you identify with:**
( ) Female
( ) Male
( ) Other

2. **Your age (in years): _____**

3. **State you live in:**
( ) Acre
( ) Alagoas
( ) Amapá
( ) Amazonas
( ) Bahia
( ) Ceará
( ) Distrito Federal
( ) Espírito Santo
( ) Goiás
( )Maranhão
( ) Mato Grosso
( ) Mato Grosso do Sul
( ) Minas Gerais
( ) Pará
( ) Paraíba
( ) Paraná
( ) Pernambuco
( ) Piauí
( ) Rio de Janeiro
( ) Rio Grande do Norte
( ) Rio Grande do Sul
( ) Rondônia
( ) Roraima
( ) Santa Catarina
( ) São Paulo
( ) Sergipe
( ) Tocantins

 © 2022. The Author(s).

4. **What is your education level? (select the highest)**
   ( ) Illiterate
   ( ) Incomplete high school
   ( ) Complete high school
   ( ) Incomplete higher education
   ( ) Complete higher education
   ( ) Postgraduation degree
5. **What is your household monthly income?**
   ( ) Less than 1 minimum wage (less than R$1,100.00)
   ( ) Between 1 and 2 minimum wages (between R$1,100.00 and R$2,200.00)
   ( ) Between 2 and 3 minimum wages (between R$2,200.00 and R$3,300.00)
   ( ) Between 3 and 4 minimum wages (between R$3,300.00 and R$4,400.00)
   ( ) Between 4 and 5 minimum wages (between R$4,400.00 and R$5,500.00)
   ( ) More than 5 minimum wages (more than R$5,500.00)
6. **When you need dental care, you are most often seen:**
   ( ) In the public sector
   ( ) In the private sector
   ( ) In both sectors

II- SELF-PERCEPTION OF ORAL HEALTH

1. How often do you feel pain or discomfort in your teeth at these times?

a. When drinking cold water and/or ice cream (cold foods in general):

( ) Never ( ) Rarely ( ) Sometimes ( ) Often ( ) Always

b. Speaking of windy environments, or with very cold air conditioning:

( ) Never ( ) Rarely ( ) Sometimes ( ) Often ( ) Always

c. When having a hot drink (e.g., coffee or tea):

( ) Never ( ) Rarely ( ) Sometimes ( ) Often ( ) Always

d. When ingesting acidic foods or drinks (e.g., cola, orange, pineapple, lemon, citric fruit juice, or salad dressed with vinegar or lemon):

( ) Never ( ) Rarely ( ) Sometimes ( ) Often ( ) Always

e. When brushing your teeth:

( ) Never ( ) Rarely ( ) Sometimes ( ) Often ( ) Always

 © 2022. The Author(s).

2. How often do you:

a. Consume acidic foods or drinks (e.g., citrus fruits, salad with vinegar or lemon, coke or other soft drinks, sports drinks, energy drinks, or alcohol):

( ) Never ( ) Rarely ( ) Sometimes ( ) Often ( ) Always

b. Fell stressed and/or anxious:

( ) Never ( ) Rarely ( ) Sometimes ( ) Often ( ) Always

c. Grind your teeth (sliding teeth over each other) and/or clench them together:

( ) Never ( ) Rarely ( ) Sometimes ( ) Often ( ) Always

d. Press the tongue against the teeth:

( ) Never ( ) Rarely ( ) Sometimes ( ) Often ( ) Always

e. Bite lips, cheek, or tongue:

( ) Never ( ) Rarely ( ) Sometimes ( ) Often ( ) Always

f. Chew on objects such as pens, paper clips, staples, etc. and/or bites nails:

( ) Never ( ) Rarely ( ) Sometimes ( ) Often ( ) Always

g. Brush your teeth vigorously:

( ) Never ( ) Rarely ( ) Sometimes ( ) Often ( ) Always

h. Use a toothbrush with medium or hard bristles:

( ) Never ( ) Rarely ( ) Sometimes ( ) Often ( ) Always

3. Do you have any of these symptoms?

a. Difficulty opening your mouth upon waking up:

( ) Never ( ) Rarely ( ) Sometimes ( ) Often ( ) Always

b. Headaches:

( ) Never ( ) Rarely ( ) Sometimes ( ) Often ( ) Always

c. Neck pain or tension:

( ) Never ( ) Rarely ( ) Sometimes ( ) Often ( ) Always

d. Pain near the ears:

( ) Never ( ) Rarely ( ) Sometimes ( ) Often ( ) Always

 © 2022. The Author(s).

4. When you feel this kind of pain, what do you do?

a. First wait to see if the pain goes away:

( ) Never ( ) Rarely ( ) Sometimes ( ) Often ( ) Always

b. Take some medicine to see if the pain goes away:

( ) Never ( ) Rarely ( ) Sometimes ( ) Often ( ) Always

c. Use any product for sensitivity without guidance from a dentist:

( ) Never ( ) Rarely ( ) Sometimes ( ) Often ( ) Always

d. Schedule an appointment with a dentist:

( ) Never ( ) Rarely ( ) Sometimes ( ) Often ( ) Always

5. How often do you brush your teeth?

( ) More than three times a day

( ) Thrice a day

( ) Twice a day

( ) Once a day

( ) I do not brush my teeth

6. Do/did you have any lesions similar to these on any of your teeth?

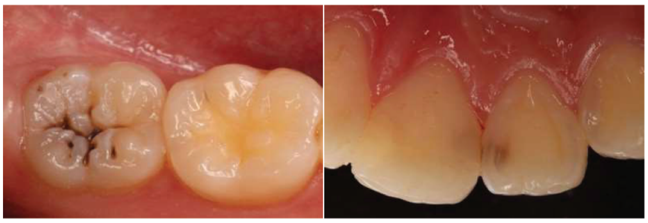

( ) Yes

( ) No

( ) I do not know

7. Do/did you have any lesions similar to these on any of your teeth (near the gums or in the biting area of the posterior teeth)?

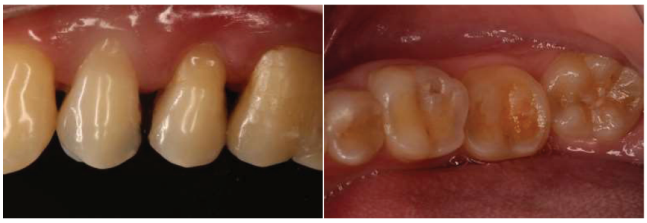

( ) Yes

( ) No

( ) I do not know

III- IMPACT OF PREVIOUS DENTAL TREATMENTS

1. Do you use or have you ever used any type of orthodontic device/braces (a device to move your teeth)? (Do not consider the possible pain/discomfort of the orthodontic movement itself).

( ) Yes

( ) No

2. Have you ever had your teeth professionally cleaned (in the office) and felt any pain/discomfort after the treatment?

( ) Yes

( ) No

( ) I do not remember

IV- PREVENTION AND TREATMENT

1. Did you know that tooth sensitivity can be prevented?

( ) Yes

( ) No

2. How do you believe it can be prevented?

a. With the guidance of a dentist

( ) Yes ( ) No ( ) I do not know

b. Performing dental procedures in the office or at home

( ) Yes ( ) No ( ) I do not know

c. With the cessation of the habits that cause the pain

( ) Yes ( ) No ( ) I do not know

d. With improved oral hygiene and nutrition

( ) Yes ( ) No ( ) I do not know

3. Did you know that tooth sensitivity can be treated?

( ) Yes

( ) No

4. What ways do you think are possible to treat sensitivity?

a. Using specific toothpastes

( ) Yes ( ) No ( ) I do not know

 © 2022. The Author(s).

b. Using lasers

( ) Yes ( ) No ( ) I do not know

c. Using orthodontic appliances/braces

( ) Yes ( ) No ( ) I do not know

d. Having professional products applied by the dentist

( ) Yes ( ) No ( ) I do not know

5. You believe this treatment is:

( ) Definitive

( ) Temporary

( ) I do not know

6. Do you use any specific product (e.g., toothpastes) for tooth sensitivity?

( ) Yes

( ) No

( ) I do not know

7. If yes, who recommended it?

( ) Dentist

( ) Friend of family member

( ) I saw it in the media (TV advertisements and/or magazines, social media such as Facebook, Instagram, Twitter, etc. or radio)

( ) Other communication vehicle

 © 2022. The Author(s).
